# “When I Was Circumcised I Was Taught Certain Things”: Risk Compensation and Protective Sexual Behavior among Circumcised Men in Kisumu, Kenya

**DOI:** 10.1371/journal.pone.0012366

**Published:** 2010-08-25

**Authors:** Thomas H. Riess, Maryline M. Achieng’, Samuel Otieno, J. O. Ndinya-Achola, Robert C. Bailey

**Affiliations:** 1 School of Public Health, University of Illinois at Chicago, Chicago, Illinois, United States of America; 2 Research Care and Training Program, Center for Microbiology Research, Kenya Medical Research Institute, Kisumu, Kenya; 3 Department of Medical Microbiology, University of Nairobi, Nairobi, Kenya; University of Cape Town, South Africa

## Abstract

**Background:**

Male circumcision has been shown to reduce the transmission of HIV from women to men through vaginal sex by approximately 60%. There is concern that men may engage in risk compensation after becoming circumcised, diminishing the benefits of male circumcision.

**Methods and Findings:**

We conducted qualitative interviews with 30 sexually active circumcised men in Kisumu, Kenya from March to November 2008. Most respondents reported no behavior change or increasing protective sexual behaviors including increasing condom use and reducing the number of sexual partners. A minority of men reported engaging in higher risk behaviors either not using condoms or increasing the number of sex partners. Circumcised respondents described being able to perform more rounds of sex, easier condom use, and fewer cuts on the penis during sex.

**Conclusions:**

Results illustrate that information about MC's protection against HIV has disseminated into the larger community and MC accompanied by counseling and HIV testing can foster positive behavior change and maintain sexual behavior.

## Introduction

Male circumcision (MC) has recently become a significant component of human immunodeficiency virus (HIV) prevention efforts after the results of three randomized controlled trials (RCT) demonstrated that circumcised men have an approximately 60% less chance of becoming infected with HIV through unprotected vaginal sex. [1]–[3] Following the publication of results from the three RCTs the Joint United Nations Programme on HIV/AIDS (UNAIDS) and the World Health Organization (WHO) recommended that MC be recognized as an intervention for HIV prevention in countries with low MC rates, high HIV prevalence, and where heterosexual sex is the main route of transmission. [4]

Despite WHO and UNAIDS recommendation, and the promotion of MC as an HIV prevention method in sub-Saharan Africa, questions remain about whether promoting MC as an HIV prevention intervention will result in decreases in HIV incidence. A key concern is that promoting MC may lead circumcised men to develop a false sense of complete protection against HIV and engage in risk compensation by halting or decreasing previous protective behaviors such as condom use or partner reduction, causing the protective effects of MC to be reduced or negated. [5], [6]

Risk compensation is the notion that individuals modify their behavior in response to real or perceived changes in risks. Pinkerton has defined risk compensation as “any behavioral change that acts to offset a reduction in risk resulting from other changes”. [7] Risk homeostasis theory has been used to describe this phenomenon. [8] Accordingly, sexual risk compensation in the context of MC is characterized by someone knowing that they are less likely to contract HIV and responding to this reduced risk by increasing behaviors associated with greater risk of HIV transmission, such as not using condoms, increasing the number sexual partners, increasing the frequency of higher-risk sex (e.g. anal sex versus vaginal sex), or increasing the frequency of sex with high-risk partners (e.g. sex workers).

The terms risk compensation and sexual disinhibition have been used interchangeably in the public health literature. The concept of sexual risk compensation implies that a change in potential harm has occurred and is in turn being countered by a change in one's level of risk behavior. Sexual disinhibition on the other hand denotes that some sort of restraint has been removed, and subsequent behavior is not related to a change in the level of risk, but rather a lowering or removal of such a moderator. The effect of alcohol illustrates disinhibition since it may lower the restraints of certain behaviors and can result in people engaging in sexual behavior that they may be less likely to perform when not inebriated, but the risks of contracting HIV related to sex remain constant. MC lowers the probability of men contracting HIV and any increase in risk behavior attributable to this known protection should be considered risk compensation. A preference for the term risk compensation, rather than disinhibition, has been expressed by Hogben and Liddon [9] and Cassell et al. [5]

Data from the three African RCTs show varying evidence of changes in sexual risk behavior after circumcision. The Ugandan RCT reported no risk compensation among participants while the South African trial reported an increase in the number of sexual encounters between 4 and 21 months after circumcision but no decline in condom use or increase in the number of sex partners. [1], [3] The Kenyan trial reported a decrease in risk behavior among both the circumcised and uncircumcised groups, with the uncircumcised men reporting less unprotected sex and greater condom use at 24 months follow-up. [2] Additionally, a subgroup of 1,319 Kenya RCT participants were recruited for a study examining risk compensation over a 12-month period using an 18-item behavioral risk propensity scale and found a significant reduction in sexual risk behavior in both uncircumcised and circumcised men, and no statistically significant difference in the incidence of sexually transmitted infections (STIs) or sexual behavior in the two groups. [10]

A prospective cohort study conducted from 2002 to 2004, before the dissemination of RCT findings, looked at sexual risk behavior following MC in western Kenya and found no differences between circumcised and uncircumcised men in the average number of risky sex partners per month or the frequency of condom use over a 12-month follow-up period. [11]

Given the variability of results from studies examining sexual risk compensation in HIV vaccine trials, [12] HIV prevention interventions, [7] STI prophylaxis, [13] antiretroviral therapy, [14], [15] and condom promotion programs, [16] and the dearth of research on sexual behavior change related to MC, the objectives of this study were to: 1) explore how MC influences sexual risk perceptions and behaviors and 2) identify and describe individual level factors that could facilitate or reduce sexual risk behaviors related to male circumcision. In this paper we characterize the sexual perceptions and behaviors of circumcised men and provide context for sexual behavior change related to MC among men in Kisumu, Kenya.

## Methods

Between March and November 2008 we conducted individual in-depth qualitative interviews and focus group discussions among women and circumcised and uncircumcised men to explore sexual risk perceptions and behaviors related to male circumcision. For the purpose of this paper only interviews conducted with circumcised men were used. Men who were recently circumcised provided first hand accounts of their pre- and post-circumcision sexual behaviors. Men circumcised in their youth provided information on whether learning that circumcision is protective against HIV influenced their sexual risk behaviors.

### Setting

Data for this study were collected in Kisumu, Kenya, the country's third largest city, and the capital of Nyanza Province. Kisumu's population of approximately 400,000 inhabitants is made up predominately of people belonging to the Luo ethnic group. It is estimated that 85% of Kenyan men are circumcised but Luo men do not traditionally circumcise. [17] Nyanza province has the lowest rate of male circumcision in Kenya at 46.7% and the highest HIV prevalence in the country at 15.3%, more than double the national average of 7.4%. [17] It is estimated that 16.9% of Luo men in Nyanza province are circumcised, [18] whereas the ethnic groups that inhabit the area immediately surrounding the Luo in Nyanza Province (i.e. Kisii, Luhya, Nandi, Kipsigis, Kuria, and Maasai) regularly practice male circumcision.

At the time of this study there were several ongoing HIV prevention activities in Kisumu as part of non-governmental organizations' (NGO) programs or research, including: counseling and testing, antiretroviral therapy, STI treatment, male circumcision, and outreach programs. Additionally, from 2002 to 2006 Kisumu was the site of a RCT that examined male circumcision's effect on HIV incidence. During the course of our study the Kisumu RCT clinic continued to function as a male circumcision, STI, and HIV counseling and testing clinic. MC services were also provided by a sexual and reproductive health NGO in Nyanza Province from June 2007 through December 2008.

### Sampling and recruitment

Respondents were recruited from community settings using purposive sampling methods. [19] To be eligible for the study, respondents needed to be 18–35 years of age, circumcised, have had vaginal or anal sex in the past 12 months, reside in Kisumu District, and be willing to be audio recorded. A screening questionnaire was used to identify eligible respondents. Recruitment locations included: shopping centers, health clinics, circumcision clinics, and on the street. Interview participants were also recruited through snowball sampling methods, whereby respondents were asked to identify potential participants who may be sources of information on sexual behavior related to MC. [20] A diverse sample of circumcised men was recruited in order to explore a wide variety of sexual perceptions and behaviors related to male circumcision.

### Qualitative data collection, management, and analysis

All project staff received training on human subjects and completed the Collaborative Institutional Training Initiative online training course on human subjects protection. The study was approved by the Institutional Review Boards of the University of Illinois at Chicago, USA and the Kenyatta National Hospital, Nairobi, Kenya.

After obtaining written informed consent, individual interviews were conducted in Dholuo, Kiswahili, and/or English, using a semi-structured interview guide designed to elicit the following information: knowledge and beliefs about MC; knowledge of MC's relationship to HIV and STIs; reasons for getting circumcised; changes in sexual activity after getting circumcised and/or learning of MC's protective affect against HIV; communication with others about circumcision; negative effects of being circumcised; how circumcision is viewed within their ethnicity's culture; knowledge and beliefs about HIV; a description of a recent sexual encounter; knowledge and opinions of HIV risk reduction strategies; and condom use. Demographic information was collected via a 35-item questionnaire at the end of the interview. To ensure confidentiality, individual interviews were conducted in a private room. Interviews lasted between 40 and 120 minutes and were audio-recorded. All interviews were conducted anonymously and no names or contact information were collected. Respondents were reimbursed 150 Kenyan Shillings (approximately US$2.25) in cash at the end of the interview for their time.

Audio recordings were transcribed verbatim in the language of the interview, and then translated into English, if necessary. All transcripts were verified by a second member of the research team against the audio recording for accuracy and any discrepancies were reconciled. A collaborative codebook was developed, whereby three members of the research team developed codebooks independently, using questions and probes from the interview guide as well as patterns, interactions, and themes that emerged from the first 13 interviews. All codes from the three codebooks were evaluated for their relevancy and merged into a single codebook. New codes were developed over the course of the study as new themes and perspectives emerged. Project staff coded the interviews using Glaser and Strauss' constant comparative method. [21] Fifty percent of the transcripts were coded by two members of the research team and were then reconciled between the coders to come up with a mutually agreed upon coding scheme. The remaining fifty percent of transcripts were coded by one member of the research team. Inter-rater reliability scores were not computed. The transcripts were imported into Atlas ti qualitative analysis software and coded electronically for analysis.

After coding the interview transcripts using Atlas ti software analytical memos related to composite codes were written. Analysis focused on understanding the significance of MC, identifying changes in risk behavior after circumcision, and factors contributing to behavior change. Discussions among the research team were held to reach consensus on data interpretations and resolve discrepancies.

## Results

Table 1 presents respondent demographic characteristics. Among the 30 circumcised male respondents interviewed (N = 30) the median age was 25. Most respondents were not married and not living with a sexual partner (63%), had completed primary school (90%), worked in the service industry (e.g. transportation or security guard) (50%), reported earning 2,000 to 10,000 Kenyan Shillings (approximately US$30–$150) per month (70%), and were from the Luo ethnic group (83%). Among respondents' sexual behaviors, 60% reported having one sexual partner in the past 30 days; 73% said they had 2 or more sexual partners in the past year, 67% said that they used a condom during their last sexual encounter, 1 (3%) reported being HIV positive, and none reported having sex with men in the past 12 months.

**Table 1:** Demographic characteristics of respondents (N = 30).

Characteristics	No. of Respondents	% of Respondents
Age (years)		
18–22	8	27
23–27	13	43
28–33	9	30
Marital Status		
Married living with spouse	11	37
Not married, not living with sex partner	19	63
Highest education level completed		
Did not finish primary school	3	10
Primary school	9	30
Secondary or vocational	10	33
Beyond secondary	8	27
Occupation		
Professional	4	13
Health worker	4	13
Service worker	15	50
Student	2	7
Unemployed	3	10
Merchant	2	7
Average monthly income		
<2,000 Shillings	6	20
2,000–5,000	12	40
5,001–10,000	9	30
>10,000	2	7
Missing	1	3
Ethnic group		
Luo	25	83
Luhya	2	7
Kisii	2	7
Kamba	1	3
Age at circumcision		
<11	3	10
11–15	2	7
16–20	10	33
21–25	8	27
26–30	6	20
31–33	1	3
Number of years circumcised		
<1	7	23
1	9	30
2–5	6	20
6–10	3	10
>10	5	17
Number of sex partners in past 30 days		
0	7	23
1	18	60
2	3	10
3	0	0
4	2	7
Number of sex partners in past year		
1	8	27
2–4	19	63
5 or more	3	10
Used condom during last sexual encounter		
Yes	20	67
No	10	33

Respondents were circumcised between 1986 and 2008. A majority of respondents were circumcised from 16 to 30 years of age (80%). The youngest age at the time of circumcision was seven years old and the oldest was thirty-three. The period of time that respondents had already been circumcised ranged from 2 weeks to 22 years, the median being one year. Of the 30 circumcised men, 11 were circumcised at the Kisumu RCT's clinic (average number of years having been circumcised = 2.4, range = <1–6 years), 10 were circumcised at the sexual and reproductive health NGO's clinic in Kisumu (average number of years having been circumcised = 0.8, range = <1–2 years), 7 were circumcised in hospitals throughout Kenya (average number of years having been circumcised = 10.4, range = <1–22 years), and 2 were circumcised in ethnic group ceremonies (average number of years having been circumcised = 16.4, range = 14–19 years). Twenty-three (77%) of the respondents were circumcised in Kisumu.

### I. MC's influence on sexual behavior

We classified sexual behavior change into three categories: 1) adopt protective sexual behaviors, defined as reducing the number of sexual partners or increasing condom use, 2) maintain same behavior, and 3) increase in risk behaviors (risk compensation), defined as increasing the number of sexual partners or reducing condom use. Table 2 lists the frequency of responses for sexual behavior change categories and circumcision venue.

**Table 2:** Sexual behavior changes after circumcision or learning that circumcision reduces transmission of HIV (N = 30)*.

	Circumcised at RCT Clinic		Circumcised at Sexual & Reproductive Health NGO clinic		Circumcised at Kenyan Hospital		Circumcised at Ethnic Ceremony		Total	
	No.	%	No.	%	No.	%	No.	%	No.	%
Adopted protective sexual behavior:										
Reduced number of partners (includes abstinence)	3	27	2	20	0	0	0	0	5	17
Increased condom use	5	45	1	10	0	0	0	0	6	20
Maintained same sexual behavior	4	36	4	40	7	100	2	100	17	57
Increased sexual risk behavior:										
Increased number of sexual partners	0	0	4	40	0	0	0	0	4	13
Decreased condom use	1	9	0	0	0	0	0	0	1	3

*Categories are not mutually exclusive as two respondents reported multiple protective behaviors, therefore not all column totals add up to 100%.

#### Adopting protective sexual behaviors

Five respondents reported reducing the number of sex partners they had and six increased their condom use after circumcision. They described changes in their sexual behavior in relation to their knowledge, attitudes, and beliefs about MC and HIV counseling and testing.

A respondent who was circumcised to lower his risk of contracting HIV had been taught about partner reduction during pre-circumcision counseling, and subsequently reduced the number of girlfriends he had.

Now I have reduced bad behavior that I had. I didn't like my behavior before I was circumcised. I liked girls, so when I received these teachings, some skills and knowledge, which I didn't have. I realized that I was messing up. I could lose my life. So that is why I decided to change. … I had two girlfriends in the past. … I took one. (18 year-old circumcised less than one year)

A respondent who took his first HIV test as part of pre-circumcision counseling explained that it was not just the circumcision procedure that changed his behavior but also undergoing HIV testing that served as an impetus to changing his behavior.

**Respondent**: … it's not just circumcision but it involves a lot about the test … before I was circumcised I used to have many girls, you could go [have sex] with many girls. Nowadays I always stick to somebody, to just one person.

**Interviewer**: Is that because you're circumcised or because of something else?

**R**: Yeah, because circumcision also changed my behavior. … So I think with circumcision, it's not specifically circumcision that makes me to behave that way, but it's like the test I'm taking. So, I always want to remain negative, like that. So it does change but not specifically that because I'm circumcised. Maybe my sex life has changed but what I'm saying is that it is because of my status. (24 year-old circumcised three years)

An 18-year-old man who did not use condoms explained that he learned how to use condoms during pre-circumcision counseling and began using them.

**I**: So the five women that you mentioned that you had sex with since [being circumcised], so how many of those did you use condoms with?

**R**: All.

**I**: … And the three women before you were circumcised how many?

**R**: I was not using.

**I**: … So why did you start using now, after you were circumcised?

**R**: I learnt that condom helps to prevent some diseases. The other days I was not, I didn't learn, but nowadays I learnt that it helps prevent some diseases. (18 year-old circumcised one year)

#### Maintaining same behavior after circumcision

Seventeen men reported maintaining their sexual behavior after getting circumcised. Seven of these men reported getting circumcised in a hospital and two in tribal ceremonies where MC was not being promoted as an HIV prevention intervention and counseling was not provided. The other eight men did receive counseling, with four circumcised at the RCT's clinic and four circumcised at the NGO clinic.

Despite wanting to get circumcised because he believed women preferred circumcised men, a RCT participant reported that the number of sexual partners he had did not change after getting circumcised.

**I**: …did you think that your sex life would change after you got circumcised?

**R**: Not really, I was just going to maintain it. So it has just remained the same. It's not that now I'm circumcised so I have to do it [have sex] quite often, no. It has not changed. (23 year-old circumcised three years)

The man elaborated further acknowledging that he feels more protected against HIV, but that it did not cause him to change his sexual behavior.

**I**: What about just like your susceptibility to STIs or HIV, do you feel any different about that?

**R**: Yeah, I do feel that now I'm less susceptible to contracting them than when I was uncircumcised.

**I**: So how does that affect your behavior then?

**R**: It doesn't affect it. It doesn't affect my behavior much. Because now I won't do it [have sex] just anyhow because I know I'm less susceptible. No. I still maintain, I'm still cautious just like I used to be even when I was not circumcised. Because we were made to understand … that circumcision will not eliminate totally but it will only reduce the chances. But we were also told that when, if you combine circumcision and maybe the use of condoms that will be a good combination in reducing your chances of getting HIV/AIDS. (23 year-old circumcised three years)

The 20 year-old man quoted below who was circumcised at the sexual and reproductive health NGO's clinic explained that being circumcised would not cause him to increase the amount of unprotected sex he had. When asked whether he viewed HIV differently after getting circumcised he replied:

… even say the girls who, that I have sex with, or those I have been having sex with in the past, most of them I'd use a condom. So now, because of AIDS, I can't say now that I am circumcised, and because it was said that it reduces the rate of infection a bit, I can just have sex with somebody without protection. (20 year-old circumcised less than one year)

One respondent, who was circumcised because he felt that it would reduce his chances of getting HIV, carried on his risky behavior after getting circumcised. He continued not using condoms, because a bishop had told him that the lubrication used in condoms develops into HIV.

**I**: … did the fact that you didn't use condoms, did that influence your decision to want to get circumcised at all?

**R**: Yeah. That one also influenced me to getting circumcised, cause when I hear that now there's an alternative.

**I**: An alternative to what?

**R**: Cause, the version about condoms is that they are, they prevent somebody from getting infection. And also concerning circumcision is that it reduces the risk of somebody getting infected. So you see circumcision was kind of an alternative of using a condom for somebody who has always had a negative attitude towards the usage of condoms. See it was kind of an alternative.

**I**: So after you were circumcised did … you ever think you were going to use them [condoms]?

**R**: I never even imagined about them. Not at all. (21 year-old circumcised three years)

#### Risk compensation

Five respondents reported engaging in riskier sexual behavior after getting circumcised. One man stopped using condoms temporarily after getting circumcised and four men reported an increase in the number of sexual partners, with no change in condom use. All five men were circumcised as part of MC programs for HIV prevention with four being circumcised at the sexual and reproductive health NGO's clinic and one at the RCT's clinic.

A respondent who was circumcised at the sexual and reproductive health NGO's clinic reported having a temporary “testing” period of unprotected sex with his wife after being circumcised. He was aware that circumcision only provided partial protection against HIV but wanted to try sex without a condom before returning to his pre-circumcision practice of using condoms occasionally with his wife to prevent pregnancy and consistently with two other sexual partners.

**R**: I did not want condoms when testing. … What's needed is just the skin against skin. … Because with testing, there shouldn't be anything that acts as a barrier.

**I**: And were you using condoms before circumcision?

**R**: Yeah, I was using.

**I**: And after circumcision have you used?

**R**: Yeah, now after that test, I just went back to normal. (27 year-old circumcised one year)

A man who was circumcised at the RCT's clinic found that being circumcised made him a more desirable sex partner. He continued to have unprotected sex with his primary girlfriend but reported using condoms with his two new girlfriends. The three other respondents who reported increasing the number of sex partners after circumcision also reported using condoms with their new partners.

**I**: … after you're circumcised what's different in your life or your behavior, or anything?

**R**: It can increase the number of girlfriends.

**I**: So how many do you have?

**R**: Three.

**I**: … And how many did you have before you were circumcised?

**R**: One. (20 year-old circumcised one year)

### II. Knowledge of MC's protective affects

#### Understanding of circumcision's partial protection

Nearly all respondents expressed an understanding that MC only offers partial protection against HIV and STIs. While only a few respondents could give an accurate figure on the percentage of protection that MC provided, there was widespread understanding that it was not 100% protection, and that other means of HIV prevention should be used in conjunction with circumcision. Men reported that they needed to continue to use condoms, or for men who practiced condom use intermittently, to increase their condom usage, be faithful to one partner, or reduce the number of sexual partners in order to maintain a low-level of HIV risk.

A respondent who heard about the benefits of MC after the publication of the results of the Kisumu RCT was circumcised at the sexual and reproductive health clinic as an HIV prevention measure. He remained concerned about HIV and felt that circumcision gave him some insurance against condom failure.

Like the other thing I would say is that I'm less worried in a way. You know even though HIV/AIDS is there but since I have that in mind, that I have 60% protection, at least it gives me some hope, because the issue of HIV/AIDS nowadays is a worry to everybody. …You know even using condoms at times, you never know you might not use a condom properly and you might get infected, so at least I know that if you have 60% to some extent you are a bit safer in a way. (28 year-old circumcised one year)

A Luhya respondent was circumcised eight years earlier in a hospital as part of his ethnic group's customs. His belief that MC did not provide total protection against HIV was informed by his knowing circumcised men who had been infected with HIV.

**I**: … since you're a circumcised man do you think that you might at one point-.

**R**: Not use a condom? It depends now. But to me I think I cannot risk. I can only risk it after marrying my wife, maybe. … But I cannot do it because I'm circumcised now. And I know the risk maybe are lesser compared to somebody who is uncircumcised. I cannot risk.

**I**: But have you thought about that before?

**R**: I've thought about it but to me it's null and void. … You know it's not good because there are some who have been circumcised but they're still getting the HIV/AIDS. That's the reason. (24 year-old circumcised eight years)

A Luo respondent who had been circumcised in a Nairobi hospital mentioned that, since the benefits of MC were not known when he was circumcised, he used condoms as a way to prevent HIV and STIs. He had since learned about the benefits of MC against HIV, but still expressed his vulnerability to HIV as being similar to that of someone who is not circumcised.

**R**: In fact when I got circumcised, the issue, the relationship between circumcision and HIV and AIDS was not very clear. That was back in 2001.

**I**: … how did you think being circumcised might change your sex life though?

**R**: … you know that when you get circumcised, as in after getting to learn the relationships about it, in fact has really made me to know that I'm no better than someone who is not circumcised in relation of HIV and AIDS and other STI infections. …what I'm saying is that if someone is circumcised and someone is not circumcised, if they still engage in risky sexual behavior without condoms with someone who is infected with the HIV and AIDS they will still get it. (29 year-old circumcised eight years)

Responses demonstrate that widespread communication about the benefits and risks of MC were taking place and that they go beyond individual counseling. The quotes illustrate that information about MC's partial protection against HIV is widespread among respondents who were circumcised in different settings and at various points in time. Respondents reported obtaining information about MC through multiple sources, including the media, community health organizations, HIV prevention programs, schools, peers, and MC programs.

#### HIV counseling and testing and its impact on behavior

MC was accompanied by HIV counseling and testing for 21 respondents. The amount of counseling received varied according to when and where respondents were circumcised. The 11 respondents who were circumcised at the RCT clinic reported receiving on-site pre- and post-circumcision counseling and HIV testing, visiting the clinic between 1 to 3 times before being circumcised and from 13 to 38 times after being circumcised for trial follow-up visits and medical care. The ten respondents circumcised at the sexual and reproductive health NGO's clinic were informed of the protection that MC provides against HIV by a recruiter during recruitment. The nine men circumcised at hospitals or in ethnic group ceremonies did not report receiving pre- or post-circumcision counseling either because circumcision was not being offered as an HIV prevention intervention, or for seven respondents, they were circumcised prior to WHO's advocacy of MC.

Among those who underwent counseling and/or testing as part of their circumcision, some credited this experience with changing their previous high-risk behaviors. Getting circumcised served as a gateway to knowledge about HIV prevention and transmission and one's HIV status. Testing HIV negative during pre-circumcision counseling was a relief for some respondents given their past risky sexual behaviors. After getting a negative test result some men expressed a sense of having a fresh start and a desire to stop or reduce their risky behaviors in order to remain HIV negative. The nine respondents who did not undergo counseling as part of their circumcision did not report protective sexual behavior change or risk compensation.

A respondent who was circumcised as a participant in the Kisumu RCT expressed how getting circumcised educated him about HIV and STIs. After he tested HIV negative he wanted to make sure that he would maintain his serostatus so he decided to remain with one sexual partner as a way to reduce his risk behavior. The HIV test also made him more concerned about his health, which drove him to start using condoms more frequently.

I: … after circumcision, did your sexual practices change?

R: Of course, I became more careful. … I know that I'm HIV negative. Also, I really want to be safe and like be, for as long as it will take. It changed me, it did. (29 year-old circumcised six years)

The same respondent spoke about increasing condom use because of counseling, but after a period of using condoms most of the time he went back to using condoms occasionally because he was monogamous and to prevent pregnancy

**I**: … after circumcision did you use condoms more or less?

**R**: More. … I used to [use condoms] but not very much …. And then when I came here [to the RCT clinic] I got more enlightened on these issues to do with HIV/AIDS and stuff of that sort, STIs. So I became more concerned about my life. So I make them [condoms] a priority, in fact during those times [while participating in the trial] I used to use condoms so much. (29 year-old circumcised six years)

### III. Other MC facilitated changes

Some men reported that the anatomical change of removing the foreskin facilitated changes in their behavior and their physical vulnerability to HIV. Men reported it was easier to use condoms, that cuts on the penis during sex no longer occurred, and that they were able to have more rounds of sex. The term rounds of sex was used by respondents to describe consecutive sex acts, that end in male ejaculation, with one partner during a single encounter. For example, if a man and woman have sex where the man ejaculates two times in the same sexual encounter that would be considered two rounds of sex.

#### Ease of condom use

Several respondents reported that condom use was easier after getting circumcised. Men described their circumcised penis as smooth and pointed and said that since there was no longer the need to pull the foreskin back to put on condoms, it was easier to use them.

… when you're not circumcised wearing a condom takes a lot of time. And then you know, normally you find that maybe that when you're wearing that condom and you take a lot of time, you find you're losing some erection. As compared to when you're circumcised, it's very easy to wear a condom. (27 year-old circumcised four years)

#### Reduced cuts on foreskin

Six men reported the elimination of cuts on their penis during sex after being circumcised. Men were not certain as to what caused the cuts and tears when they were uncircumcised but after getting circumcised and no longer getting cuts they attributed the cuts to the movement of the foreskin during sex. Respondents expressed an awareness that the cuts made them more susceptible to HIV and STIs and were satisfied that circumcision had stopped the cuts from occurring.

**I**: … before you were circumcised were there injuries on the foreskin when you were having sex?

**R**: … Yes. … You may sometimes find that it is broken and bleeding.

**I**: And does that happen nowadays?

**R**: … No, no.

**I**: … it has reduced or it doesn't happen at all?

**R**: It hasn't happened to me. (18 year-old circumcised less than one year)

#### Increased number of rounds of sex

Four respondents reported that after being circumcised they increased the number of rounds of sex and were capable of having one to four additional rounds. In all instances men attributed the increase to the fact that they had been circumcised since it coincided with the procedure.

… I can say that I've also improved cause the number of rounds initially two then you are just gone completely. But now I can manage even up to three. I can manage up to three or four. You see, in a night. (21 year-old circumcised three years)

## Discussion

The results of this study illustrate that MC does not necessarily lead to risk compensation, and that there are different behavior change outcomes. Previous studies exploring risk compensation associated with MC have been quantitative in nature, conducted during an RCT, and have provided limited detail about the context of risk compensation and protective sexual behaviors. Our study conducted among RCT participants and men circumcised in other venues provides details of motivations for changing or maintaining sexual behaviors after circumcision. This study helps in understanding individual sexual risk perceptions and behaviors that may change during the scale-up of MC programs. Our results underscore the need for HIV prevention counseling to be integrated with MC services.

A majority of men reported adopting protective sexual behaviors or not changing their sexual behavior after getting circumcised, or after learning of MC's protective effects against HIV. Several factors were associated with adopting protective behaviors: becoming aware of one's sexual risk behavior, HIV and MC counseling and education, HIV testing, and a desire to remain HIV negative. Some respondents reported that MC accompanied by pre-circumcision HIV counseling and testing influenced them to adopt protective sexual behaviors, including reducing high-risk behavior. In other instances counseling seems to have made men aware that some of their past behaviors increased their risk of contracting HIV, which influenced them to adopt risk reduction strategies. All men who mentioned HIV counseling and testing as influencing their behavior had gone through a MC program established for HIV prevention purposes. However, respondents who engaged in risk compensation also received counseling.

Respondents who maintained their sexual behavior may have established limits that they thought were effective in avoiding HIV infection, such as using condoms consistently or being faithful to one partner, and may have resolved to continue with the same sexual practices. We also observed an inverse form of risk compensation from a man who acknowledged high-risk sexual behavior and got circumcised in order to reduce his chance of acquiring HIV. It is likely that other men may not feel the need to change their high-risk behavior because, by being circumcised, they decrease their chances of contracting HIV and therefore will maintain previous sexual behavior. Given the varied sexual behaviors reported by study participants, our results support the guideline that counseling should be tailored to the individual. [22]

All respondents who did not attend pre- or post-circumcision counseling maintained their sexual behaviors. These men were more likely to be circumcised at a younger age, before their sexual debut, in hospitals, or as part of ethnic group customs. They did not report receiving risk reduction counseling and did not discuss the process of getting circumcised as instrumental in changing their sexual behavior, which may be because these men established HIV risk reduction routines earlier in their sexual lives prior to knowing that MC was protective against HIV. Importantly, they reported being aware of MC's partial protection against HIV, suggesting that information on MC was reaching the general public and may be effective in mitigating risk compensation.

The fact that little risk compensation was reported may be due to several factors, including the effects of counseling, HIV testing, knowledge that MC only offers partial protection, and condom availability. All respondents who demonstrated risk compensation were circumcised at clinics that offered MC as an HIV prevention measure roughly one year before their interviews, and received some counseling before getting circumcised. The respondent who reported sex without a condom after getting circumcised also indicated that this sexual behavior was similar to his pre-circumcision behavior. The men who reported increasing the number of sexual partners after circumcision reported using condoms consistently with their new partners, potentially offsetting some of the risk of having concurrent partners. In light of these findings, MC and HIV counseling and testing programs should work to identify individuals who are disposed to engage in risk compensation during counseling sessions by asking men about their plans to change their sexual behavior following circumcision and emphasizing that MC only offers partial protection.

Behavior change facilitated by the physical and mechanical aspects of removing the foreskin may also have implications for HIV and STI prevention. Respondents reported the ability to increase the number of rounds of sex after being circumcised, which could potentially lead to greater exposure to HIV and STIs through an increased frequency of sexual acts. In the Orange Farm, South Africa trial, the investigators reported an increase in the number of sexual encounters, but no change in the number of sex partners. [1] This may reflect an increase in the number of rounds per partner as suggested by the circumcised participants in our study. Despite the greater number of encounters in the circumcised men, there was still a 60% reduction in HIV with circumcision in the South African trial, as well as a reduction in some other STIs. [1]

Other reported benefits of circumcision among respondents were that it made condoms easier to use and eliminated penile cuts. This is consistent with findings from the Kisumu RCT cohort in which circumcised men had a 39% reduction in the odds of reporting bleeding, cuts, scratches, or abrasions on their penis acquired during intercourse. [23] Reducing or eliminating such penile trauma could be a secondary benefit of male circumcision in reducing the risk of HIV transmission. Also of importance is that some men found it easier to use condoms after being circumcised, which may encourage them to use them more frequently.

Responses illustrate that behavior change is a dynamic process and examining sexual risk behaviors is not a linear exercise. Circumcised men may not just adopt positive behaviors or only increase risk behaviors. Men who reduced the number of sexual partners to one may in turn stop using condoms with that partner. We also saw that respondents who increased the number of concurrent sexual partners subsequently increased condom use with their new partners. That MC may play a role in promoting an increase in concurrent sexual partners is important to recognize since concurrent partnerships have been shown to be instrumental to the spread of HIV in sub-Saharan Africa. [24], [25] Also, if these new partners become longer term partners, condom use is likely to wane, making concurrent partnerships a significant HIV risk. [26]–[29] Since MC is not a stand-alone HIV prevention intervention it is vital that messages emphasizing continued condom use and partner reduction accompany MC programs and services.

While counseling and testing was reported by respondents as being instrumental in influencing their behavior, the content and frequency of the counseling provided to circumcised men was not a primary area of inquiry of our study. Therefore we have limited data describing what components of counseling were effective in bringing about behavior change. This is an area that merits further research. Nevertheless, since boys and men in various settings in sub-Saharan Africa are likely to be circumcised for many reasons, in addition to HIV and STI prevention, and counseling can have an impact on sexual risk behavior, as seen in our study, (see also Kamb et al. [30] and Denison et al. [31]) counseling should be made available with MC services. In settings where MC is not provided as an HIV prevention measure it is unlikely that hospital staff and ethnic group circumcisers are trained to provide HIV counseling. Counseling could be realized by providing training to clinicians and traditional circumcisers, placing counselors in clinical and traditional circumcision ceremony settings, or establishing referral systems between MC settings and HIV counseling and testing clinics.

The findings from this study should be viewed within the context of the following limitations. The results are based on self-reported experiences. People are sometimes reluctant to share personal information regarding their sexual behaviors, so it is possible that respondents withheld information or fabricated answers during the interviews. Recall bias also may have affected responses. It is possible that some of the men we interviewed were not circumcised since we relied on respondents' reports of their circumcision status. However, since uncircumcised men were eligible for the study, there was no reason for respondents to lie about their circumcision status. Eleven of the men were participants of the Kisumu RCT examining male circumcision's protective affect against HIV and were tested for HIV and received HIV prevention counseling prior to getting circumcised and after circumcision every six months for two years. Such intense and repeated counseling is unlikely to be representative of the level of counseling other adult men being circumcised receive. This research was conducted in the early stages of scaling up MC, so respondents in our study may have been those most motivated to seek protection offered by MC and more motivated to avoid risk compensation. Additionally, because this research was carried out in western Kenya mostly among the Luo, an ethnic group that traditionally does not circumcise yet are surrounded geographically by circumcising groups, these results may not be applicable in other countries or regions where circumcision is less widely practiced.

Despite these limitations, several important findings emerged from our data. Respondents have illustrated that protective behavior change can result from men being circumcised. Counseling placed within or related to MC programs appears to be influential in promoting protective behavior change among participants. We conducted interviews with respondents who had been circumcised for less than a year up to 22 years, and it is promising to see that men circumcised for longer periods of time exhibited similar sexual behavior and circumcision knowledge to those more recently circumcised, because it shows that information about MC is reaching the general population. In addition to the direct benefit circumcision provides in reducing HIV transmission there may also be secondary benefits for individuals such as reduction in penile cuts during sex and easier condom use, resulting in lower risk of HIV infection.
